# MicroRNA-874 inhibits growth, induces apoptosis and reverses chemoresistance in colorectal cancer by targeting X-linked inhibitor of apoptosis protein

**DOI:** 10.3892/or.2022.8280

**Published:** 2022-02-07

**Authors:** Jinfeng Han, Zhongmin Liu, Nanya Wang, Weiyun Pan

Oncol Rep 36: 542-550, 2016; DOI: 10.3892/or.2016.4810

Following the publication of this paper, it was drawn to the Editors attention by a concerned reader that certain of the western blotting data shown in Fig. 5D were strikingly similar to data appearing in different form in other articles by different authors. Owing to the fact that the contentious data in the above article had already been published elsewhere prior to its submission to *Oncology Reports*, the Editor has decided that this paper should be retracted from the Journal. After having been in contact with the authors, they agreed with the decision to retract the paper. The Editor apologizes to the readership for any inconvenience caused.

